# A Two-step Technique for Neo-umbilicoplasty in the Abdominal Reconstructive Population

**DOI:** 10.1097/GOX.0000000000002341

**Published:** 2019-07-25

**Authors:** Ledibabari M. Ngaage, George Kokosis, Bartlomiej Kachniarz, Rachel Pedreira, Erin M. Rada, Arthur J. Nam, Jonathan Pearl, Stephen Kavic, Yvonne M. Rasko

**Affiliations:** From the * Division of Plastic Surgery, Department of Surgery, University of Maryland Medical Center, Baltimore, Md.; † Department of Plastic and Reconstructive Surgery, John Hopkins University School of Medicine, Baltimore, Md.; ‡ Johns Hopkins University School of Medicine, Baltimore, Md.; § Department of Surgery, University of Maryland Medical Center, Baltimore, Md.

## Abstract

The umbilicus is the centerpiece of the abdomen and an important cosmetic landmark. Secondary intention healing can be used to create the illusion of an umbilicus in reconstructive surgery. However, there is a sparsity of literature on the role of neo-umbilicoplasty in this subset of patients and how they perceive their new umbilicus. We conducted a retrospective review of patients who received a neo-umbilicus during an abdominal reconstructive operation in 2016–2018. The umbilicus was amputated intraoperatively and tacking sutures were placed to create an indentation in the soft tissue; an inverted-V incision was sutured to create the illusion of an umbilicus. Postoperatively, patients were asked to rate their satisfaction with umbilicus appearance on a 5-point Likert scale. Of the 10 patients included; the majority were female (9:1), with a mean age of 37 years (range: 26–50) and mean BMI 29 (range: 21–38). The most common procedure performed at time of neo-umbilicoplasty was hernia repair (80%). There were no wound complications associated with the neo-umbilicus. Satisfaction was high (median 5, range: 1–5) and independent of time since surgery. Three patients gave scores below 5 and cited reasons of “lack of indentation” (n = 3, scores 4, 4, and 1), and “insufficiently notable scar” (n = 1, score 1). We hereby describe a simple surgical technique for neo-umbilicoplasty in patients undergoing reconstructive abdominal surgery that leads to long-lasting cosmetic satisfaction. Further studies of patient-reported outcomes and refinement of the technique will maximize the aesthetically pleasing results.

## Abstract 2

Supplemental Digital Content is available in the text.

## INTRODUCTION

The umbilicus is an important cosmetic landmark and its distortion can be a source of distress. It is well documented in the aesthetic literature that many consider creation of a new umbilicus, also termed neo-umbilicoplasty, a natural component of abdominoplasty, the main purpose of which is to create an aesthetically pleasing abdomen.^1–3^ It is an accepted fact that amputation of the umbilical stalk is sometimes inevitable to prevent vascular compromise of the umbilicus, particularly in cases that require abdominal wall reconstruction.^4,5^ In such cases, umbilicoplasty is not an option, necessitating a choice between no reconstruction and creation of a neo-umbilicus. Nevertheless, there is little discussing neo-umbilicoplasty in the reconstructive population or how these patients perceive their new umbilicus.

There is no clear consensus on a “gold standard” neo-unmbilicoplasty technique.^6^ Secondary intention healing has the benefit of no donor site morbidity unlike other techniques and is thought to mimic the natural scar of the umbilicus.^1^ This study will describe our technique for neo-umbilicoplasty in the reconstructive population and evaluate patient-reported outcomes.

## METHODS

We conducted an Institutional Review Board-approved retrospective review of patients who received a neo-umbilicus during an abdominal reconstructive operation between 2016 and 2018 by a single plastic surgeon at a tertiary medical center. Postoperatively, patients were asked to rate their satisfaction with the appearance of their new umbilicus by an independent physician using a 5-point Likert scale.

### Operative Technique

A standard abdominoplasty approach was used to elevate a subcutaneous abdominal flap superiorly and resect excess tissue. The location of the neo-umbilicus was along the midline and at the approximate height as the original as agreed by surgeon and patient.

**Fig. 1.**
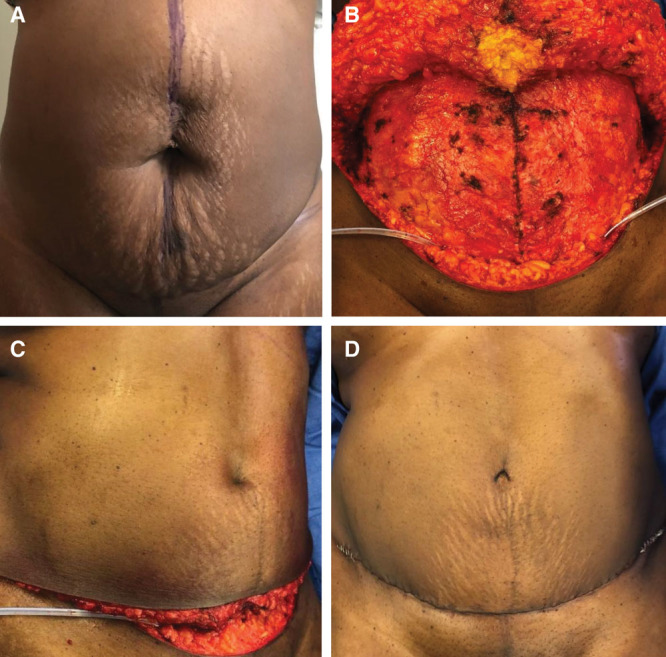
A, Frontal preoperative photographs of a patient demonstrating supraumbilical and umbilical hernia in the setting of a wide diastasis. B, Following umbilicus amputation and repair of diastasis recti, coring out of subcutaneous tissue and a tacking suture is placed at the original umbilicus site to create an indentation. C, Intraoperative lateral view following creation of indentation, placement of bilateral Jackson-Pratt drains and reapproximation of superior and inferior skin flaps; indentation of the abdominal wall from the umbilical scar can be seen. D, Frontal image of abdomen after skin closure and placement of external scar. The inverted-V umbilical incisional scar. We found that a high number of gut sutures has led to a degree of reactive scarring that best mimics an umbilical scar.

First, the umbilicus was resected in cases of extensive deformity, involvement in hernia, poor umbilical stalk vascularity, or to minimize hernia recurrence risk according to patient preference (Fig. 1A). All fascial defects were repaired with figure-of-eight polydioxanone suture (PDS). Any diastasis of the rectus abdominis fascia was then repaired using interrupted Ethibond sutures. Umbilical reconstruction required 2 steps. First, the umbilicus was reconstructed by first creating an indentation (Fig. 1B). Subcutaneous fat is cleared with a conical taper and 2–3 interrupted PDS sutures are placed to anchor the deep dermis to the abdominal fascia. The sutures were closely spaced and included a deep dermal component to create a well-defined indentation in the abdominal skin surface (Fig. 1C). PDS suture was used given its relatively slow absorption rate.

Second, an inverted-V skin incision was made to create the illusion of an umbilical scar. A 1–2 mm partial-thickness strip of skin was excised and closed with interrupted 5-0 Chromic gut sutures (Fig. 1D). The abdominoplasty incision was then closed in standard fashion over 2 Jackson-Pratt drains. An abdominal binder was placed at the conclusion of the procedure and a Xeroform dressing is placed postoperatively for 7 days. The patients are counseled to wear the binder for 4 weeks.

## RESULTS

Ten patients were included. The majority were females (9:1), with a mean age of 37 years (range: 26–50) and mean BMI 29 (range: 21–38). Their demographical information is detailed in Supplementary Digital Content 1. (See table, Supplemental Digital Content 1, which displays patient characteristics, *http://links.lww.com/PRSGO/B143*.)

Median number of previous abdominal surgeries was 2 (range: 0–5). The most common procedures performed at time of neo-umbilicoplasty were hernia repair (80%). There were no complications associated with the neo-umbilicus. Additionally, none of the patients required a reoperation. Mean follow-up time was 182 ± 227 days (range: 42–786). Illustrative preoperative and postoperative images can be viewed in Supplementary Digital Content 2. (See figure, Supplemental Digital Content 2, which displays frontal and lateral photographs taken ten months following surgery. The healed inverted-V scar can be seen, *http://links.lww.com/PRSGO/B144*.)

The median satisfaction rating was 5 (range: 1–5) and independent of time since surgery. Three of the 10 patients who scored their aesthetic outcome with satisfaction scores of less than 5, reported lack of indentation (n = 3 patients, scores 4, 4, and 1), and insufficiently notable scar (n = 1, score 1). Of note, the same patient scored the lowest score (1) in the above.

## DISCUSSION

The aesthetics of the umbilicus are a common concern in surgical planning as it affects appearance of abdominal contour and can dictate patient satisfaction.^7^ Previous studies have assessed the hypothetical aesthetics of an optimum umbilicus,^8,9^ but few have evaluated the aesthetics of a neo-umbilicus from a patient perspective. The majority of patients achieved an aesthetically pleasing outcome. We therefore advocate for the use of this simple technique as a quick (approximately 10 minutes) and effective way to attain high patient aesthetic satisfaction.

The surgical approach can be divided into 2 key concepts: (1) indentation (Fig. 1B, C) and (2) creation of an external scar (Fig. 1D). The literature reports that a protruding umbilicus is less appealing than a concave appearance,^9,10^ a notion supported by our findings. In our study, all patients who were not completely satisfied with their umbilicus cited “lack of indentation” as the most common reason for dissatisfaction. Indeed, some surgeons offer secondary reconstruction for patients who feel their umbilicus is too shallow.^11^ It is of note that the least satisfied patient possessed a BMI less than the mean (BMI < 27). In such patients, there is little surrounding fat from which to create a central indent.

Although the preferred umbilicus appearance is reported as an oval shape with superior hooding, to form a “T,”^8,9,12,13^ in our study, we found that the majority of patients achieved high satisfaction with our inverted-V scar. The inverted-V incision is designed to give a scar notch and hooding to mimic the umbilical anatomy.^14^ This shape was chosen as it has a reduced risk of vascular compromise (versus a round incision) and optimal cosmesis (appearance of hooding).^15^ There is no consensus as to which neo-umbilicoplasty approach would be most appropriate.^6^ However, secondary intention healing has the benefit of speed and simplicity with a minimal risk of dehiscence and hernia recurrence.

This study is limited by its small sample size, retrospective nature, and relatively short follow-up. Further limitations of study are described in the Supplementary Digital Content.

## CONCLUSIONS

We present a simple surgical technique for neo-umbilicoplasty in patients undergoing abdominal wall reconstruction with umbilical stalk amputation. Our technique utilizes indentation and secondary wound healing to lead to high cosmetic satisfaction. Further studies of patient-reported outcomes and refinement of the technique to meet these demands will maximize the aesthetically pleasing results.

## Supplementary Material
