# Supplementary material for: A Two-step Technique for Neo-umbilicoplasty in the Abdominal Reconstructive Population
Source: Plast Reconstr Surg Glob Open. 2019 Jul 25;7(7):e2341. doi: 10.1097/GOX.0000000000002341 (PMC6952142; doi:10.1097/GOX.0000000000002341)
Supplement: Supplementary file 1 [file gox-7-e2341-s001.pdf]

SDC 1

SDC 1. Patient characteristics

Patient	Gender	Age (years)	BMI	Number of pregnancies	Bariatric surgery	Previous umbilical hernia	Number of prior abdominal surgeries	Procedures
**1**	M	40	35.5	N/A	Y	Y	2	Ventral hernia repair<br>Panniculectomy
**2**	F	40	31.0	3	N	N	4	Ventral hernia repair<br>Abdominoplasty<br>Diastasis recti repair
**3**	F	27	27.7	1	N	Y	1	Umbilical hernia repair<br>Diastasis recti repair
**4**	F	50	33.3	0	Y	N	1	Panniculectomy<br>Diastasis recti repair

**5**	F	30	27.4	2	N	N	0	Umbilical hernia repair<br>Diastasis recti repair
**6**	F	38	21.3	2	N	N	2	Umbilical hernia repair<br>Diastasis recti repair
**7**	F	35	37.7	2	N	N	1	Umbilical hernia repair<br>Panniculectomy<br>Diastasis recti repair
**8**	F	32	23.8	3	N	N	2	Umbilical hernia repair<br>Diastasis recti repair
**9**	F	54	26.5	2	N	N	2	Liposuction for body contouring s/p peritoneal mesothelioma
**10**	F	26	29.8	2	N	Y	5	Panniculectomy<br>Ventral hernia repair
