# Supplementary figures and images for: A Two-step Technique for Neo-umbilicoplasty in the Abdominal Reconstructive Population
Source: Plast Reconstr Surg Glob Open. 2019 Jul 25;7(7):e2341. doi: 10.1097/GOX.0000000000002341 (PMC6952142; doi:10.1097/GOX.0000000000002341)

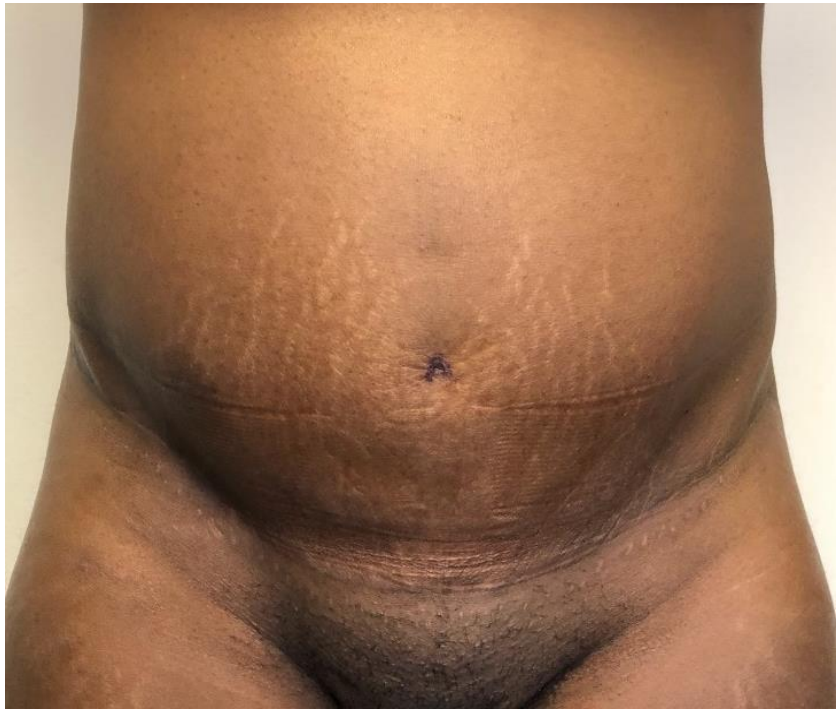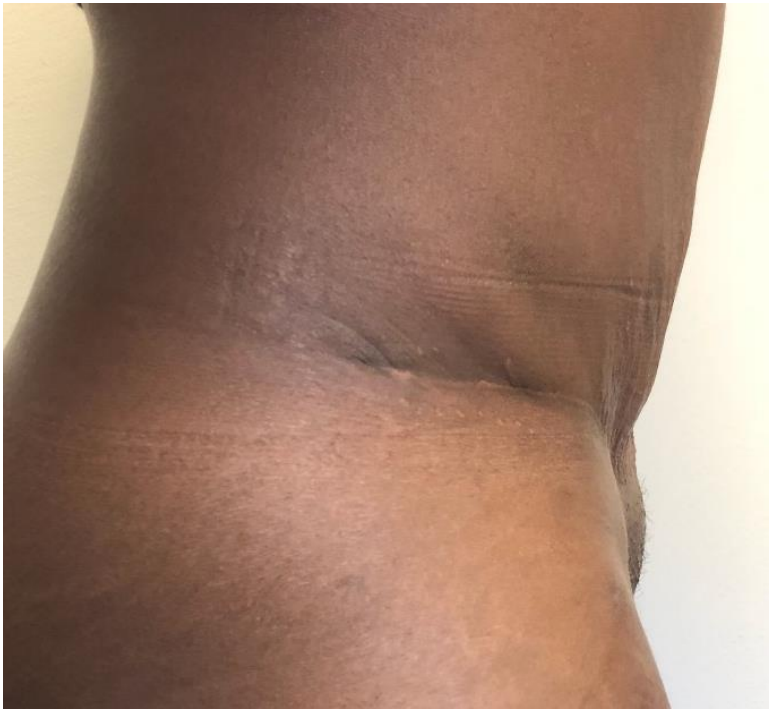

Supplement: Supplementary file 2 [file gox-7-e2341-s002.pdf]
